# Conscious Causality, Observer–Observed Simultaneity, and the Problem of Time for Integrated Information Theory

**DOI:** 10.3390/e26080647

**Published:** 2024-07-30

**Authors:** John Sanfey

**Affiliations:** Independent Researcher, London, UK; johnsanfey@mac.com

**Keywords:** consciousness, time, quantum mind, mind-matter relationship, observer–observed simultaneity, electromagnetic theories of consciousness, integrated information theory, causality, meaning: the hard problem, abstract realism

## Abstract

Without proven causal power, consciousness cannot be integrated with physics except as an epiphenomenon, hence the term ‘hard problem’. Integrated Information Theory (IIT) side-steps the issue by stating that subjective experience must be identical to informational physical structures whose cause-and-effect power is greater than the sum of their parts. But the focus on spatially oriented structures rather than events in time introduces a deep conceptual flaw throughout its entire structure, including the measure of integrated information, known as *Φ (phi)*. However, the problem can be corrected by incorporating the temporal feature of consciousness responsible for the hard problem, which can ultimately resolve it, namely, that experiencer and experienced are not separated in time but exist simultaneously. Simultaneous causation is not possible in physics, hence the hard problem, and yet it can be proven deductively that consciousness does have causal power because of this phenomenological simultaneity. Experiencing presence makes some facts logically possible that would otherwise be illogical. Bypassing the hard problem has caused much of the criticism that IIT has attracted, but by returning to its roots in complexity theory, it can repurpose its model to measure causal connections that are temporally rather than spatially related.

## 1. Introduction 

Causality is so fundamental in physics that there is seldom any need to mention it. The principle is simply embedded in the structure of laws and theories that describe observable phenomena. Spacetime is structured as continuous motion without simultaneous causation; a cause is always in the past of its effect and every observable effect must have a cause. The dimensional relationships in spacetime vary with the motion of observers within it, but for quantum systems, things are very different. Here, time is a rigid framework, external to the system along with observers. But the framework ensures that quantum mechanics follows the same temporal sequencing, or causal ordering rule with respect to causality. No causal influence or information can ever go back in time. Time may be treated differently in each branch of physics, but both retain causal ordering, which is the key to resolving the problem of conscious causation. 

Consciousness is unobservable, so if it has causal power, it must be implemented by a corresponding, observable physical state. However, it follows that the physical state must be capable of achieving the same cause-and-effect outcomes without someone having conscious experience at all. This is ‘the hard problem’ for which no recognised solution exists [1,2]. One phenomenal feature of consciousness that has not attracted much attention is that the experiencer and experienced occur simultaneously; they are not separated in time [3]. Consciousness is often described as there being ‘something it is like’ to see red, or to feel pain, etc., [4], a definition that assumes phenomenal simultaneity but without recognising its significance to the hard problem. But simultaneous causation is not allowed anywhere in physics, either classical or quantum, so any proof that Observer–Observed Simultaneity (OOS) has causal consequences would constitute a breakthrough in terms of the hard problem. 

Integrated Information Theory (IIT) bypasses the subtleties of the hard problem by simply stating that consciousness has causal power because it exists, a claim it justifies by assuming the reversed Eleatic principle is equally true as Plato’s original version [5,6]. Plato had said that if something has causal power, it must exist [7]. But IIT claims the reverse, that ‘if something exists, it must have causal power’ [6], and, consequently, subjective consciousness must be identical to an informational physical state whose causal power is greater than the sum of its parts. The theory is built around this psycho-physical identity, but in doing so, it misses an opportunity to develop insights into the metaphysical problems of time, causality, and observation. Resolving the hard problem is likely to be of interest to everyone concerned with fundamental reality. 

IIT has undoubtedly advanced the science of consciousness considerably, not least by attempting to explain conscious causation. But it has also attracted much criticism. I will show that many of the theory’s recognised flaws stem from bypassing the hard problem. The central identity in IIT ensures that the theory focuses on observable structures in the brain, and the mathematics required to quantify the informational cause-and-effect power of their input and output connections. Among the criticisms is the logistical difficulty of measuring these quantities, but here I will show that this and many of the conceptual problems would disappear by recognising the importance of Observer–Observed Simultaneity (OOS) in the phenomenon of consciousness. IIT began its journey in the 1990s by focussing on complexity [8]. Along the way, it shifted from the temporal dynamics of complex patterns to spatially orientated cause-and-effect structures, but spatial models of causality tend to eventually violate the temporal sequencing that is so fundamental to physical causality. By returning to its temporal roots in complexity, IIT can correct many of its problems and become more capable of achieving its ultimate goal of mathematically mapping conscious phenomenology to an underlying substrate. 

The arguments here will show that for intelligent systems not experiencing the simultaneity intrinsic to ‘something it is like’, certain concepts are logically impossible, which has observable consequences for the behaviour of the system [3]. If true, it follows that consciousness can be part of the same physics that describes all phenomena, and that it can be produced by physical systems. However, it also follows that since the additional degrees of freedom are derived from phenomenal simultaneity, they are unobservable and unpredictable by any temporally sequential process. Consciousness is consequently indistinguishable from a strongly emergent phenomenon as a matter of principle. But that does not mean that conscious events in the brain do not create observable signatures. It is perfectly possible to detect both the consequences and antecedent processes associated with OOS and to correlate them with phenomenological content in conscious subjects. Although these signatures must occur at timescales measured in microseconds, the technology required to detect them already exists. However, the software required to study complexity at these timescales remains under-developed [9].

In the next section, I will review some of the criticism that the conceptual foundations of IIT have attracted. Then, I will summarise the OOS arguments and introduce a new argument that explores the relationships between time, consciousness, and causality. Finally, I will describe some thoughts on how IIT might shed much of its conceptual and logistical baggage by incorporating OOS and its supporting hypotheses. If adopted, IIT would no longer need to identify the causal structures specified by its postulates, or unfold their cause-and-effect structures, or then identify the structure most likely to have the highest impact. The theory could ditch all this and simply quantify and correlate what happens to information flows associated with detectable OOS events in the brain. 

## 2. Published Critique of IIT’s Conceptual Structure

### 2.1. Overview of IIT’s Structure

IIT is based on five axioms that describe the phenomenology of consciousness. Each axiom is paired with a postulate in accordance with the identity between subjective experience and informational cause-and-effect structures. The postulates specify the physical substrates necessary to instantiate each axiom [10,11,12,13]. The latest version of IIT, version 4.0, introduced the *zeroth* axiom as a foundational principle, which states that the existence of consciousness is more certain than anything else [5].

The first of the five axioms is *intrinsicality*, namely, that ‘every experience is for an experiencer’, in other words that subjective consciousness is completely private, inaccessible to outside observation. Its corresponding postulate is that a physical substrate has intrinsic causal power, in other words, it has cause-and-effect power on itself, independently of outside factors and greater than the sum of its parts. 

Subsequent axioms and their corresponding postulates complete the description of conscious phenomenology in IIT. Each experience is *composed* of phenomenal distinctions. Consciousness is *informational*, and information forms an *integrated* whole. The fifth axiom–postulate pairing is *exclusivity*, which holds that the maximally integrated cause-and-effect repertoire of each informational structure is specific for each experience. The amount of integrated information in a system is known as *Φ (phi)*, and when an information state is maximally irreducible in terms of its cause-and-effect power the information state is conscious and can be quantified as *Φ^max^*. 

### 2.2. Weakness in the Foundations of IIT

Axioms in mathematics are truths established by proof, whereas in science, they can be just widely accepted. IIT’s axioms are neither. The dubious reversal of the Eleatic principle has already been discussed, but IIT’s axiomatic foundation is not widely accepted either, because as Bayne pointed out, some respected philosophers claim that consciousness is an illusion [14]. Bayne also questions whether the postulates are derived deductively, or even whether they are the best abductive inferences to explain the axioms. Frankish challenges IIT’s claim that the axioms capture the phenomenology of consciousness on the basis that subjective introspection is notoriously unreliable, and cites the example that humans subjectively experience the sun rotating around the earth [15]. 

The authors of IIT regard their axioms as irreducible starting points [5], which is the sort of description usually reserved for metaphysical first principles, namely those axioms that can neither be deduced from, nor reduced to, more basic principles and which concern reality at its most fundamental level. The theory’s axiom–postulate structure is founded on the principle that the existence of physical reality is the best explanation for what we experience [12,13]. Most people would agree with this statement, but the problem for IIT is that belief in physicalism is just one of several logically possible options, including solipsism, none of which can be proven false by any means. Choosing between physicalism and non-physicalism is a personal choice, but it has causal consequences for the person concerned, which means that subjectivity has causal degrees of freedom for which non-physical (idealist) explanations are logically possible [3]. For these reasons, IIT can never be considered fundamental in the metaphysical sense of describing the mind–matter relationship by first principles at the deepest level. 

Mørch highlights a particular logical contradiction in IIT’s intrinsicality axiom–postulate. She points out that if conscious causal power is objectively inaccessible when subjectively experienced but objectively measurable as brain structures, then the relationship between them cannot be an identity [16]. This logical contradiction has not yet been addressed by IIT and is another consequence of conceptualising causality in spatial rather than temporal terms. 

### 2.3. Integrated Information, Φ (phi)

Merker and colleagues challenge IIT’s concept of integrated information. They argue that IIT fails to show why *Φ (phi)* is constitutive of consciousness per se, and not merely a property shared by all efficient systems such as integrated power grids, human social networks [17], or biological immune systems [18]. The cerebral cortex conducts many operations requiring high *Φ* that are not conscious. Merker and colleagues are particularly critical of IIT’s lack of attention to the phenomenon of first-person perspective even though it is conceptually central to the notion of subjective intrinsicality. However, IIT is making attempts to rectify this issue [19]. 

Krohn and Ostwald identified a problem with the exclusion axiom–postulate pair. They showed that multiple, maximally irreducible cause-and-effect repertoires (MICs) exist for any given quale of experience, which should lead to overlapping experiences in contradiction of the exclusion principle. They called this problem ‘quale under-determinism’ [20], and suggested that choosing the MIC with the lowest number of elements, or lowest dimensionality would best satisfy IIT’s principle of minimal multiplication. At first, the creators of IIT made the opposite choice, namely, the MIC with the highest dimensionality repertoire [13]. But Moon offers a novel solution, which is to choose an MIC repertoire that makes a difference, which he terms the ‘difference-making criterion’ [21]. Moon’s idea seems to have hit home with the creators of IIT. Subsequent versions of the theory introduce the Intrinsic Difference (ID) measure, which has become an important part of the theory [5,22,23]. The ID measure identifies which of the possible MIC repertoires maximises the information ‘received and sent correctly *from and to* the system’ and is most likely to be the one chosen by the system [5,23]. This move by IIT is conceptually consistent with the logic of the theory, but measuring ID requires not only knowing the structural inputs and outputs in the substrate for conscious experience, but also evaluating which output is most likely to have the highest impact. With more than 100 trillion synaptic connections in the brain, it is difficult to see how the ID measure might make the theory operational in practice. Conceptually, the ID measure reinforces the theory’s dependence on spatially related structures and amplifies the logistical nightmare of actually measuring *Φ* in living brains. 

### 2.4. Panpsychism as Pseudoscience

In IIT, the maximally irreducible informational structure ***is*** the quale of experience, not something equivalent or correlated to it [6]. For this reason, IIT has always admitted to being a panpsychist theory, which is the idea that consciousness should be considered a fundamental feature of reality. In IIT, the entities carrying the property of consciousness are complexes of elements rather than fundamental particles. Consciousness is not a function of these physical complexes, it ***is*** the complexes [10]. This important distinction led a prominent group of researchers to describe IIT as unscientific, even as pseudoscience [15,24,25]. The group highlighted the implausibility of IIT’s claim that a grid of connected logic gates could be conscious by virtue of their informational configuration, even when they were functionally inactive, a criticism applicable to all theories appealing to panpsychism. The situation might be different if IIT had made testable predictions that were quickly proven true, but that has not happened, at least no predictions that were specific to the theory [18,24].

Doerig and colleagues had earlier published an argument apparently reducing IIT’s central identity to absurdity. The *Unfolding Argument* targets the claim in IIT that consciousness depends on the nature of the informational complexes rather than their causal consequences [26]. IIT claims that consciousness is possible in artificial systems, but only when those systems have recursive or recurrent information architectures. In IIT, systems based on feedforward flows of information can never be conscious [5,10]. The Unfolding Argument is that for any conscious system satisfying IIT’s postulates, there exists a possible feedforward system that will achieve exactly the same cause-and-effect outcomes, but which IIT would define as non-conscious. This apparent absurdity led Doerig and colleagues to conclude that consciousness cannot be explained by any causal structure theory, and indeed, that all causal structure theories of consciousness must either be false or outside the realm of science. How can subjective consciousness be identical to one type of cause-and-effect structure but not to another with empirically identical cause-and-effect outcomes? Among their conclusions is that we cannot hope to develop a comprehensive theory of consciousness without specifying what consciousness does [26], a conclusion shared by Merker and colleagues [17]. To know what consciousness does, we require an explanation that satisfies the temporal sequencing of events demanded by causality in physical laws [3,27,28]. 

Hopkins and McQueen describe an argument somewhat similar to the Unfolding Argument. Their *Filled*–*Unfilled* argument is based on the logical possibility that two experiences could be identical despite having different physical substrates and different measures of *Φ* [29]. Many visual illusions occur when the brain inserts information it predicts to be missing from an image. Hopkins and McQueen used pairs of images, one of which required filling in by the brain, and the other which they altered or pre-filled beforehand. The brain experiences the same image in both cases despite each having different neural pathways, and IIT predicts a much higher *Φ* for the illusory image because it requires more recursive loops than the pre-filled image. It seems absurd that a system with low or even zero *Φ* can produce the same subjective experience as a substrate with high *Φ*. At the very least, the argument suggests that IIT’s choice of physical substrate is mistaken but alternatively, it could mean something much worse. 

### 2.5. ‘Greater Than the Sum of Its Parts’, but What Parts?

Barrett and colleagues identified a problem for IIT at the level of fundamental physics, where reality is typically described by continuous fields or other forms of vector spaces. But IIT’s fundamental identity does not define a particular level of granularity where its informational complexes of elements come into existence [30,31]. Without well-defined granularity, IIT’s informational complexes are ungrounded in physics. Barrett proposed a technical solution that would apply to electromagnetic (EM) field theories of consciousness, known as the Field Integrated Information Hypothesis (FIIH) [31]. The creators of IIT accepted Barrett’s critique but not his EM hypothesis, while noting that the question of whether fundamental reality is continuous or granular at the Planck scale remains open [32]. However, it is difficult to see the relevance of Planck scale physics (10^−35^ m, and 10^−43^ s) to the phenomenology of consciousness. IIT has begun to investigate how it might adapt its algorithms to quantum mechanics [22], but without considering whether EM fields might be more important than which neurons are producing them, or whether the underlying problem might be related to the continuum versus quantum problem in physics. 

Even if the physical structure of its fundamental complexes was well defined, the logistical challenge of measuring *Φ^max^* seems insurmountable. In its present form, IIT must first identify those sets of units that fulfil the properties required by the postulates, then unfold their cause-and-effect structures before assessing the probability that the system will transition into the state with the most impact [5]. 

There is a sense that IIT’s authors are beginning to see that all psycho-physical identities must eventually run into the same dead-end of describing consciousness as epiphenomenal. They admit that low *Φ* systems would be behaviourally indistinguishable from conscious ones [10]. Even in version 4.0 with its Intrinsic Difference (ID) measure, it is conceded that a low or zero *Φ* system could achieve the same outputs with the same causal consequences as a high *Φ* system [5], as shown by the Unfolding Argument. For a theory whose primary purpose is to explain conscious causation, this admission is damming. However, in the following sections I will show that IIT’s mathematical system can be repurposed by returning to its temporal roots in complexity theory. By amending its concept of intrinsic causal power from spatial intrinsicality to temporal simultaneity it can preserve the concept of phenomenal intrinsicality, for reasons that will become clear in Section 4.

Next, I will summarise the OOS arguments, before introducing a new argument suggesting that the fundamental duality in nature has nothing to do with mind and matter but is entirely a fact about consciousness, and that each aspect of the duality has a very different temporal ontology. I will show that this duality is metaphysically irreducible. 

## 3. Observer–Observed Simultaneity (OOS) and Conscious Causation

Observer–Observed Simultaneity (OOS) is inherent in the description of ‘something it is like’ to experience things. Experiencer and experienced are not separated by time. The following argument will show that some facts only become logically possible when OOS exists. Since simultaneous causation is not possible in physics, and since logically possible facts have causal consequences, this is sufficient to prove that consciousness has causal power, that it can be integrated with physics, and that it can be produced by physical systems [3]. The methodology is to examine what can be deduced from initial premisses that are phenomenally certain. The argument is the following:

Another way of stating IIT’s zeroth axiom is to say that all *completely certain facts* are facts about consciousness. The set of completely certain facts includes the following:

- Something is happening
- I am not consciously causing it

We cannot be certain *what* we are experiencing, only that there is something rather than nothing, and that we cannot make it disappear instantly by simply willing it so. That does not prove idealism false, only that we are not consciously causing reality right now. We cannot do anything *knowingly*, without being conscious of doing so. Next, consider an intelligence that does not experience ‘something it is like’, i.e., it does not experience redness or pain as being present. But suppose the AI is super-smart and perfectly logical. It may understand that its sensory and cognitive systems might be creating false information. However, there is nothing it can learn that could make it certain it is not causing what it perceives because logically, its observing self must reside in the same perceptual and cognitive systems that may or may not be creating false perceptions. A simultaneous observer on the other hand, knows it is logically possible that the observing self could be made of different substance to perceived reality. Simultaneous presence is the reason that solipsism cannot be proven false. When conscious presence is experienced with certainty, it becomes logically possible that mind stuff could exist and that observable reality could be the product of unconscious mind, or alternatively, that we created reality in the past and somehow forgot about it. None of these possibilities exist logically for the AI. The information provided by conscious simultaneity is uniquely available to a conscious observer, and it has causal consequences. 

Once proven that consciousness has causal power, it becomes possible for consciousness to be integrated with physics in principle, and for it to be produced by physical systems. However, the phenomenon of conscious experience remains forever private. Its additional degrees of causal freedom, being derived from phenomenal simultaneity, cannot be observed nor predicted by any temporally extended process. But the phenomenon does have observable consequences, which makes it indistinguishable from a strongly emergent phenomenon. Consciousness can be considered as exerting downward causation on the fundamental elements that constitute brain structure. 

The second argument will show how conscious causality operates within the laws of physics, by a mechanism that already exists in physics.

### 3.1. Observing Causal Motion

Matter can be defined as any observable that we are not knowingly causing right now. That changes nothing in physics, but it does mean that we can consider emotions and thoughts to be physical, like any other observable phenomenon. The term ‘subjective consciousness’ continues to describe there being ‘something it is like’ to experience something.

We know for certain that ‘something is happening’, which implies change over time. We are not knowingly causing reality, and gradually we have developed an understanding of spacetime as a continuum where everything observable is caused by something in its past. But causal ordering creates a paradox between observing and causality. The problem is as follows:

Ontologically, there are no points in a continuum. Motion is continuous, so every point in spacetime is really an infinitely divisible interval. Consequently, everything we observe is a causally structured interval whose beginning causes the end of the interval. This is true for every point in time. Therefore, since a cause is always in the past of its effect, everything we experience includes the retention of something that no longer exists. In other words, everything we experience contains change from A to B, where A is a re-creation, a representation or retention of some sort, when B exists. It is impossible to separate the process of perception from anything perceived. It follows that every observable difference from nothing must *intrinsically* reflect the process of observation. 

This is not very different to what Kant, Schopenhauer, and many others have described [3], but it highlights two important points. Firstly, there is no level of granularity of reality in which the observed does not contain something that reflects the process of observation; observer and observed always occur together. Secondly, any objective or third-person description or theory must use abstract devices that solve the same problem as human sensory perception. If they did not, the theories would be empirically unsound. Devices such as calculus and field theory exist to solve the problem that human perception solves when it creates sensory models and intervals of time. That is not to say that reality is a creation of mind. Far from it. It does mean that consciousness only sees models, either the sensory models of perception or the conceptual models of thought and theoretical physics. For sensory models, consciousness is the observer, whereas conceptual models use abstract systems that are functionally equivalent to human perception. In both cases, the observer projects forms that connect past and future moments of continuum together into a meaningful narrative. In each perspective, subjective (sensory) and objective (conceptual), there is an observer and an observed. In the former the observer is ontologically real, whereas in the latter, the observer is abstract. This subjective-objective complementarity is a philosophy described elsewhere as *Abstract Realism* [3,33], and forms the basis for explaining how consciousness implements its causal power within the laws of physics, and does so in the following way.

Matter is always a model whose past and future components are glued together in a manner that reflects human awareness. When the meaning of a model changes or is replaced conceptually, our subsequent behaviour may change but matter will continue to behave as it has for billions of years. The same mechanism operates in physics. When theoretical models change as they did when Newtonian mechanics gave way to relativity, human behaviour also changes. But matter continues to behave the same, indifferent to what humans think it to be. We can never know what matter really is, as Kant and Schopenhauer fully understood. Consciousness sees only models, not the stuff being modelled. 

The OOS arguments suggest that consciousness evolved to create uncertainty and momentary pause for thought. Simultaneity generates additional degrees of freedom in any moment, which makes space for creative thought, for new ideas to emerge and be recognised. The next, third OOS argument is new, and penetrates deeper into the relationships between time, causality, and observation. 

### 3.2. Time and Duality

Descartes’ famous ‘cogito ergo sum’ is generally considered to describe the awareness of being conscious, rather than the process of conceptual reasoning, an interpretation pre-dated by Aristotle and later by Avicenna’s floating man argument [34]. In this section I will argue something subtly different; that consciousness cannot exist without ‘awareness of awareness’, and that this requires conceptual content. The question of whether awareness is possible without any content is debatable [35]. Some have argued that being aware of awareness is pure awareness with ‘no objects of awareness’ [36], which seems to imply no content, conceptual or otherwise. The question is important for understanding how third-person theories emerge from first-person experience, an essential requirement for any theory of consciousness [37]. 

To investigate the question, consider the following thought experiment. Suppose it were possible for nothing to exist except consciousness. That cannot be imagined without creating minimal content such as an empty space. But imagination is not required; the argument is a logical one. We can just ask whether awareness could logically be present if consciousness were the only thing in existence, but we were not aware of it. The answer must be no. If the only thing that can be experienced is the phenomenon of experiencing, then not experiencing it must be identical to not experiencing anything. To put it another way, there can be no ‘something it is like’ to not experiencing awareness when the only thing experienceable is awareness. This seems to require that awareness of awareness is a primitive form of conceptual content, without which consciousness would not exist. Consider what happens when causality is introduced. 

We are not *knowingly* causing our consciousness to exist right now and cannot become instantly unconscious by willing it so. But what is causing consciousness? Perhaps surprisingly, there are idealist and realist explanations, just as there are for external reality. We might imagine that we had somehow created consciousness in the past but forgot we did so. Alternatively, we might suppose that consciousness is caused by something entirely outside the mind. Each paradigm has a very different temporal and causal ontology, despite being empirically indistinguishable. In the idealist perspective, causality and time are internal to the mind and nothing else exists, especially in this thought experiment. In the realist paradigm, on the other hand, time and causality are external. This suggests that consciousness operates as two simultaneous perspectives, experiential (subjective) and conceptual (objective), both necessary for the existence of consciousness. If true, then evolutionary pressure developed consciousness to improve sensory modelling using curiosity to codify and categorise what is perceived, and create third-person, communicable concepts, which is clearly beneficial in evolutionary terms. But if true, it follows that consciousness is what creates duality from the unitary phenomenon of experience described by William James, for example [38]. Even if consciousness were the only thing in existence, it would create duality because of uncertainty about causation. 

I will return to this later. But first, back to IIT. 

## 4. Can IIT Incorporate Observer–Observed Simultaneity, and Its Consequences?

Despite its conceptual flaws, IIT’s instincts are sound. Consciousness is indeed more certain than anything else and is the starting point for *all* knowledge. Another sound instinct is that consciousness has causal power, but the mistake was to not tackle the hard problem first and prove it so. The hard problem is intimately related to other fundamental problems in physics, and any solution will inevitably be a surprising one. 

IIT is also correct about the importance of information, but its conceptualisation of it is metaphysically flawed. To a conscious observer, everything looks like information because, as John Wheeler said, observed reality is answering questions that we and our instruments are asking of it. In that sense, the universe is participatory [39]. He went even further with his Mutability Principle by suggesting there is no conserved property that can be un-conserved, if we look hard enough, including properties such as electric charge [40]. In OOS terms, consciousness only experiences models, whether sensory or conceptual, not the stuff being modelled. 

Given all the criticism, it may seem surprising that IIT’s cause-and-effect notion of intrinsicality can be readily adapted for temporal rather than spatial intrinsicality. The intrinsic axiom can be modified to read *awareness of awareness creates uncertainty, which has observable consequences.* The word ‘observable’ is an opportunity for IIT to return to its roots in complexity theory, which would enable it to ditch much of the conceptual clutter that makes the theory inoperable in its present form and to direct its machinery instead towards the temporal causal connections that underpin consciousness. The ‘temporal intrinsicality’ of simultaneity is unobservable, but both its antecedents and consequences will have physical signatures that can be correlated with conscious experience. 

### 4.1. Back to the Future for IIT: Perturbational Complexity

Ironically, the best tool for measuring levels of consciousness in medical settings was developed in the early days of IIT. In 1998, Tononi and Edelman described conscious experience as being an *integrated* whole and highly *differentiated* [8]. Before rejecting functionalism in favour of cause-and-effect structures, they had concluded that neural processes must be both *functionally* integrated and differentiated. In 2009 the notion of perturbation was introduced, using Transcranial Magnetic Stimulation (TMS) to trigger responses in the brain whose complexity could be analysed using Electroencephalograms (EEGs) [41,42]. The algorithms of the Perturbational Complexity Index (PCI) have become sufficiently sophisticated to distinguish consciousness from unconsciousness. Ironically, the PCI approach in its present form may be better suited to competitor theories of IIT based on the Global Workspace (GW) ideas, which have a more open approach to functionalism and feedforward flows of information [43,44,45,46,47]. 

The *Φ* measure appeared in the early years of this century [48,49], when IIT was beginning its fateful shift away from statistical temporal dependencies in dynamical patterns [50], a move that proved conceptually disastrous. But IIT can easily return to its pioneering roots in functional complexity by directing its attention to studying electromagnetic patterns, especially those postulated by OOS theory, as described below. Doing so would enable the theory to ditch the logistical clutter required to identify and measure the potential causal power of structures that, in any case, appear to be irrelevant, especially if the following empirical data are to be believed. 

There is increasing evidence for the importance of electromagnetic activity in the brain [18,51]. This is hardly surprising, considering that every time a neuron sends a signal, an *electromagnetic* (EM) field is created. Pinotsis and Miller showed that working memories correlate better with patterns in EM fields than with the neurons producing them [52], which is in line with the Filled–Unfilled Argument. The authors suggest that patterns in EM activity help to organize complex spatiotemporal dynamics of the human brain and are important in cognitive processing [53,54]. It is no coincidence that PCI works in practice by detecting EM responses to magnetic stimulation.

OOS theory predicts that consciousness can be produced by physical systems, and it makes some testable predictions for how this could be achieved. The intrinsic meaning of OOS interactions may be unpredictable and unobservable in principle, but it is perfectly possible to detect when such interactions occur. 

### 4.2. OOS Predictions

The OOS arguments describe consciousness as a mirroring subjective–objective duality, unified and hidden in phenomenal simultaneity. The best hypothesis for how this might be achieved by physical systems is that consciousness is instantiated in the bi-directional interaction of two mirroring electromagnetic (EM) information systems, such that one (the observed) occurs inside the other (the observer), where the latter has immediate and widespread access to system memory [3]. 

The central idea is that information in the EM field of synchronously firing neurons can be communicated by cross-frequency resonant coupling with higher-frequency EM fields produced in microtubules where resonant patterns can be remembered. The information is thus replicated into multiple copies in each of the synchronously firing neurons. There is a growing body of evidence for the importance of EM activity inside microtubules, which is summarised elsewhere [3], but some new and exciting developments are worth mentioning here. Recently published evidence strongly supports the ability of microtubules to communicate information using quantum-entangled photons in a process called superradiance [55]. Also, a paper from Bandyopadhyay’s laboratory describes how photonic information can be preserved while being electromagnetically transduced through different frequencies of EM fields within microtubules, from the kilohertz level (10^3^ per second) at microtubular surfaces, through the megahertz level (10^6^/s) of the tubulin protein, to the gigahertz level (10^9^/s) of the inner water layer, and the terahertz (10^12^/s) molecular level [56]. Heleker has recently developed a device that appears to confirm widespread quantum activity within brains and is also developing a bi-directional theory, which may or may not be similar to what is being presented here [57]. On the question of whether OOS events are detectable, Bandyopadhyay’s laboratory has been developing advanced EEG technology with the Dodecanogram (DDG), which is already capable of detecting EM activity in the brain across 12 orders of magnitude from Hz to teraherz (10^12^/s) [9]. At present, the practical applicability of DDG is limited by the computing power required to analyse the vast amount of information condensed by transduction into the higher frequencies.

### 4.3. How IIT Might Incorporate OOS

There is nothing wrong with IIT’s emphasis on the importance of integrating information. The mistake was identifying causality with physical informational structures, which ultimately leads to logical absurdities. In doing so, the theory lost sight of how consciousness is a unified field of diverse elements experienced from a first-person perspective [17], although it has begun to correct that particular aspect [19]. One general solution to IIT’s problems, proposed by Mediano and colleagues, is that IIT should lower its sights and develop a ‘weaker’ version focussing on explanatory correlates between the dynamics of information processing and certain aspects of consciousness [58]. Others have made similar suggestions [59]. However, OOS is an opportunity for IIT to continue its pursuit of the stronger ambition of mathematically modelling the phenomenology of consciousness. 

Assuming that DDG technology becomes capable of analysing data at timescales much shorter than are consciously perceptible, and assuming that OOS is correct, there is no reason why IIT cannot adapt its conceptual structure to measuring correlations in the bi-directional EM interactions that underpin consciousness, both before and after the private phenomenon of the conscious ‘now’. The high frequencies of the interacting fields mean that the cause-and-effect relations being measured are incredibly brief. But crucially, the causal relations do not violate causal ordering in time. Temporal repurposing would satisfy one of the important early principles that characterised Tononi’s early collaboration with Edelman. *Recursiveness* is responsible for the absurdity highlighted by the Unfolding and Filled–Unfilled arguments but would not apply with temporal repurposing for the simple reason that here, recursiveness occurs in the tiny time intervals before and after conscious phenomena and, consequently, does not violate causal ordering in time. 

In the OOS hypothesis, the EM fields of the microtubules store and repeat previous patterns of resonance with synchronised firing by the neurons in which they exist, including the immediate past necessary to explain the conscious now [60,61], which may only be tens or hundreds of milliseconds in duration [62]. Neither large-scale nor long-duration quantum coherence is required in this model. Consciousness occurs when elements of system memory, multiply copied in microtubules, compete to find cross-frequency resonances with the lower frequencies of macroscopic EM fields, and manifest their content in consciousness. The hypothesis makes use of work by Bandyopadhyay and colleagues [9,56,63], and predicts that such stored patterns percolate up from stored timescales of 10^12^ Hz, through 10^9^ Hz, through 10^6^ Hz, and to 10^3^ Hz, as they compete to find new resonances with current neural synchrony. The observer’s perspective is from system memory, most of which is subconscious but primed for rapid retrieval as it competes to become phenomenally manifest in the longer timescales of conscious awareness (10^−1^ s). If IIT adopts this model, and its philosophical partner, Abstract Realism [3,33], it could set about mapping phenomenal distinctions in conscious experience to cross-frequency correlations within DDG patterns. 

## 5. Discussion

Causal ordering in time continues to be embedded in the fundamental laws of classical and quantum physics despite efforts to re-think locality and causation as demanded by quantum phenomena. Neither information nor any causally influential property such as momentum can ever go back in time. Given that simultaneous causation is impossible, it is all the more surprising that the phenomenal simultaneity in ‘something it is like’ to experience has not attracted more attention. It is the key to explaining how consciousness interacts with physical causation: the hard problem. 

The OOS approach begins from the same starting point as IIT, from the only absolute phenomenal certainty: consciousness. But, whereas IIT makes an immediate assumption that what we are experiencing is best explained by physical realism, OOS pauses and first examines what can be deduced with logical certainty without making any assumptions. If done correctly, the approach should deliver fundamental principles of consciousness. 

It is certain that something is happening, which we cannot eliminate by mental will alone. Since we are not knowingly causing existence, we cannot be certain what is causing it, which makes the idealism versus realism question logically impossible to resolve. But only an intelligence with simultaneous awareness can understand that. The solipsism problem can be side-stepped by regarding everything we cannot cause to instantly disappear as ‘physical’. In other words, physical laws function exactly the same as before, even for solipsists. Every observable aspect of the ‘something is happening’ contains an explanatory model produced by sensory perceptual systems. Sensory models have developed under evolutionary pressure to help us survive falsification by mortality [64]. All models have an infinite range of possible subjective meaning to an observer, and when meaning changes, our subsequent behaviour may change as a consequence. The same mechanism operates in physics. Conceptual explanatory models have changed from supernatural forces to general relativity and quantum mechanics, but the behaviour of matter has not changed for almost fourteen billion years.

The temporal ordering in the scientific model of causality forbids the ontological co-existence of past and future. But human perception has found an empirical solution by creating the conscious ‘now’ with its sensory contents. Whatever that solution is, it must be functionally replicated in theoretical physics; otherwise, physics could not claim to be empirically based. Conceptual models must contain abstract systems that are functionally equivalent to human perception: Abstract Realism [3,33]. This brings us to the question of how physical systems might produce conscious experience. There are many quantum approaches to consciousness; for a review, see [65]. Some approaches retain the randomness of quantum mechanics, but others reach for something fundamentally deeper than spacetime. Pauli and Jung described the relationships between entangled particles in terms of meaningful acausal correlations [66], thereby implying that ‘meaning’ might have an ontological existence at a deeper level of physical reality than spacetime, in a manner resembling panpsychism. Modern approaches follow the Holographic Principle [67], including attempts to describe spacetime as an emergent property of quantum information where spacetime is to qubits what water is to atoms [68].

It is always tempting to look for new physics to explain consciousness, especially when there is evidence that human cognition displays evidence of quantum peculiarities [69,70]. But that approach is usually a mistake. Consciousness is the only absolute certainty in existence. A better approach is to follow IIT’s example and seek to specify what consciousness does before considering how it might be explained from the existing physics toolbox. The ultimate goal of a consciousness theory is to make specific predictions from which new physics can be discovered.

OOS theory is an electromagnetic mirroring system, not a quantum ontology. The storage and retrieval of memory does require localised, multiply replicated episodes of quantum entanglement within the intra-neuronal EM fields of microtubules, but memories compete for conscious manifestation via EM fields. Consciousness is the bi-directional interaction of two electromagnetic systems, one of which can store memories indefinitely by transducing the information electromagnetically down to frequencies of 10^12^ Hz and then retrieve them by reverse transduction, where they compete to resonate again at the electromagnetic frequencies of neural synchrony. The technology already exists to detect these higher frequencies [9,56], so the hypothesis should become testable relatively soon, once computing power is sufficient to interpret the inevitable complexity of these high frequencies.

A final word on the central theme of this special edition. IIT is an important and well-constructed theory but would do well to return to its pioneering roots in complexity theory. Any theory that takes on the problem of conscious causation deserves respect, but ought to recognise the importance of causal ordering in time, a principle that has never been empirically violated. The innerness of consciousness is temporal simultaneity, and, as Doerig pointed out, structural notions of conscious causation are doomed to fail [26]. By recognising the intrinsicality of consciousness as temporal, IIT can ditch the requirement of identifying, unfolding, and assessing probabilities of spatial structures before the quantification of their causal power can even begin. Assuming the software becomes available, DDG should be able to identify hotspots of electromagnetic resonance at timescales well below the conscious now, and IIT can use its mathematical formalism to assess their causal impact and correlate them with subjective phenomenology.

## Data Availability

No new data were created or analysed in this study. Data sharing is not applicable to this article.
